# Older Latino Mental Health: A Complicated Picture

**DOI:** 10.1093/geroni/igaa033

**Published:** 2020-08-18

**Authors:** Daniel E Jimenez, David Martinez Garza, Verónica Cárdenas, María Marquine

**Affiliations:** 1 Department of Psychiatry and Behavioral Sciences, University of Miami Miller School of Medicine, Florida; 2 Moores Cancer Center, University of California, San Diego; 3 Department of Psychiatry, University of California, San Diego

**Keywords:** Mental health, Mental health treatment and prevention, Migration history, Older Latino

## Abstract

The aggregation of Latino subgroups in national studies creates an overly simplistic narrative that Latinos are at lower risk of mental illness and that foreign nativity seems protective against mental illness (i.e., immigrant paradox). This broad generalization does not hold up as the Latino population ages. Given that social inequalities for risk appear to widen with age, the social disadvantages of being Latino in the United States increase the risk for mental illness across the life span. This review focuses on the mental health of older Latinos, specifically the 3 subgroups with the longest residential history in the United States—Mexicans, Puerto Ricans, and Cubans. We examine relevant epidemiological and clinical psychopathology studies on aging in these Latino populations and present evidence of the heterogeneity of the older Latino population living in the United States, thus illustrating a limitation in this field—combining Latino subgroups despite their diversity because of small sample sizes. We address the migration experience—how intraethnic differences and age of migration affect mental health—and discuss social support and discrimination as key risk and protective factors. We conclude with a discussion on meeting the mental health needs of older Latinos with a focus on prevention, a promising approach to addressing mental illness in older Latinos, and future directions for mental health research in this population. Success in this endeavor would yield a substantial reduction in the burden of late-life depression and anxiety and a positive public health impact.

**Translational Significance:** Aggregation of Latinos into one, uniform ethnicity masks key differences in the prevalence of depression and anxiety, immigration histories, and sociopolitical difficulties that differentially affect their mental health. In order to address the mental health needs of older Latinos, it is important to keep in mind the need for cultural case conceptualizations of individual patients for the purpose of tailoring interventions to the unique circumstances and backgrounds of the patient.

The question of differences in mental health between Latino subgroups has been of interest to researchers stretching back to the early 1980s; however, previously published data on mental disorders and psychiatric symptoms between Latino subgroups remain sparse. With few exceptions, national surveys have combined Latinos into a homogeneous, monolithic group. When doing so, these show that Latinos, in aggregate, are at lower risk of all lifetime psychiatric disorders compared to non-Latino whites (Alegría et al., 2008; Breslau et al., 2006; Kessler et al., 2003). However, a much more complicated picture emerges when age and subethnicity are taken into account. This review focuses on the mental health of older Latinos, specifically the three subgroups with the longest residential history in the United States—Mexicans, Puerto Ricans, and Cubans. We discuss some of the landmark studies that have provided insight into intraethnic differences in the prevalence of depression and anxiety as well as the immigration histories, and sociopolitical difficulties that differentially affect mental health. In addition, we address social support and discrimination as important risk and protective factors in older Latino mental health. We conclude with a discussion on meeting the mental health needs of older Latinos with a focus on prevention, a promising approach to addressing mental illness in older Latinos, and future directions for mental health research in this population. Throughout the review, we make clear when the research is based on Latinos as a whole or on specific Latino groups (e.g., Mexican Americans, Cuban Americans, and Puerto Ricans).

## Differences in the Prevalence of Depression and Anxiety

Inconsistent with previous data on younger adults, Jimenez et al. (2010) found that the prevalence of depression and anxiety in older Latinos (aged older than 60 years) was equal to, and in some cases greater, compared to older non-Latino whites. In a study comparing lifetime and 12-month prevalence of the fourth edition of the Diagnostic and Statistical Manual (DSM-IV) psychiatric disorders, Latinos and non-Latino whites had similar lifetime prevalence rates of any depressive disorder (16.4% vs. 12.2%) and of any anxiety disorder (15.3% vs. 13.5%; Jimenez et al., 2010). Significantly higher 12-month prevalence rates of any depressive disorder (8.0% vs. 3.2%) and major depressive episodes (7.3% vs. 2.9%) were found in Latinos than in non-Latino whites (Jimenez et al., 2010). The Latino sample was divided into four subethnic groups—Mexican, Puerto Rican, Cuban, and other Latinos (mainly from the Dominican Republic, Colombia, El Salvador, Ecuador, Guatemala, Honduras, Peru, and Nicaragua). However, this study aggregated older Latinos into one large group and did not examine whether results varied across Latino subgroups due to limited sample sizes.

Jimenez et al. (2010) used the combined data from the three nationally representative studies included as part of the National Institute of Mental Health Collaborative Psychiatric Epidemiological Surveys (CPES): the National Latino and Asian American Study (NLAAS), the National Study of American Life, and the National Comorbidity Study Replication. These studies shared a primary objective: to collect data on the prevalence of psychiatric illness, impairments associated with these disorders, and treatment seeking for these disorders among a representative sample of adults in the United States (Pennell et al., 2004). The NLAAS sample was nationally representative of English- and Spanish-speaking residents (aged older than 18 years) in the noninstitutionalized population of the coterminous United States (Heeringa et al., 2004) and was designed to be nationally representative of the total Latino population and to allow for comparisons stratified by subethnic groups. The CPES used a modified version of the World Mental Health Composite International Diagnostic Interview (Kessler & Ustun, 2004) to identify the presence of lifetime and 12-month psychiatric disorders according to the DSM-IV (American Psychiatric Association, 2000) and International Classification of Diseases-10.

Using a slightly younger subsample of the CPES data set (aged older than 55 years), Woodward et al. (2012) estimated lifetime prevalence of mood and anxiety disorders for older non-Latino whites, Latinos, Asian Americans, African Americans, and Caribbean blacks and examined demographic, socioeconomic, and immigration correlates of those disorders. They found that older non-Latino whites and Latinos consistently had a higher prevalence of mood (13.8% and 13.9%, respectively) and anxiety disorders (16.8% and 15.2%, respectively). Furthermore, they concluded that lower risk for disorders among Latinos is more pervasive in younger cohorts and within lower education groups (Woodward et al., 2012). Similar to Jimenez et al. (2010), Woodward et al. (2012) did not examine differences between Latino subgroups. Despite this limitation, these studies—with their use of a comprehensive, fully structured diagnostic interview and a nationally representative sample—enhanced our understanding of mood and anxiety disorders across older minority groups and provided evidence of a complicated picture of mental health in older Latinos. Important differences in the prevalence of depression and anxiety may be overlooked when older and younger Latinos are lumped together.

The Hispanic Health and Nutrition Examination Survey (HHANES) conducted from July 1982 to December 1984 by the National Center for Health Statistics (NCHS) was the first nationwide population-based survey of the physical and mental health and nutrition status of the three largest Latino groups in the United States: Mexican Americans, Cuban Americans, and Puerto Ricans. Instead of a national probability sample, the HHANES targeted civilian noninstitutionalized Mexican Americans in the Southwestern United States, Puerto Ricans in New York City, and Cuban Americans in Miami, FL (NCHS, 1985). Results from the HHANES showed that older Latinos aged 65–74 years reported high levels of depressive symptomatology as defined by a score of 16 or higher on the Center for Epidemiologic Studies Depression scale (CES-D; Radloff & Wales 1977) in all three Latino groups. However, there was variation between the three groups. Puerto Ricans reported the greatest prevalence of high depressive symptomatology (27.9%), Cuban Americans had the lowest prevalence (11.3%), while Mexican Americans were in between those two groups (13.2%; Moscicki et al., 1987, 1989; Narrow et al., 1990; Potter et al., 1995). The HHANES was not specifically focused on older Latinos; therefore, the sample sizes in all three groups were low. However, it was an early indication that the burden of mental illness was quite high among older Latinos as a whole, that this burden was not equally shared among various Latino subgroups, and laid the foundation for the subsequent epidemiological studies.

Following up on the results of the HHANES, the Hispanic Established Populations for Epidemiologic Studies of the Elderly (H-EPESE) was developed to specifically focus on older Mexican Americans. The H-EPESE assessed the physical health, mental health, and functional status of older Mexican Americans and was the first large-scale population-based study that employed a representative sample of older, community-dwelling Mexican Americans from five states in the Southwestern United States (Markides et al., 1996). Using the same definition of high depressive symptomatology, Black et al. (1998) found a prevalence of 25.6%, which was substantially higher than the rate reported for older Mexican Americans in the HHANES. The prevalence of depression was also considerably higher than rates reported in studies of older non-Latinos which had used similar methodologies (Berkman et al., 1986; Blazer et al., 1991; O’Hara et al., 1985).

Similarly, the Sacramento Area Latino Study on Aging (SALSA) was a longitudinal cohort study of older, community-dwelling Mexican Americans residing in California’s Sacramento Valley who were aged 60–101 years at baseline in 1998–1999. SALSA aimed to assess cognitive, physical, and social functioning in older Latinos in Sacramento, CA. Eighty-five percent of the sample was of Mexican origin with the remainder of the participants coming from Central and South America and the Caribbean. Using the same definition of high depressive symptomatology as in HHANES and H-EPESE (i.e., CES-D ≥ 16), González et al. (2001) found a prevalence of depression of 25.4% among this predominately older Mexican American sample, which was nearly identical to what was reported from H-EPESE (25.6%; Black et al., 1998). For the first time, there was convergence from two large population-based studies indicating that more than a quarter of older Mexican Americans were experiencing major psychological distress, which was higher than what was being reported for non-Latino whites (Blazer et al., 1998). However, questions still remained surrounding the prevalence of depression as well as anxiety using diagnostic measures among older Latinos at a national level.

The Health and Retirement Study (HRS; http://hrsonline.isr.umich.edu) is a longitudinal study of a population-based sample of more than 20,000 Americans older than the age of 50. The HRS is a representative sample of U.S. adults who reside in the community and were approaching retirement, with a 2:1 oversample of African American and Latino populations. Details of the design and historical context of the HRS have been reported previously (Juster & Suzman, 1995). Using the HRS, Yang et al. (2008) examined the prevalence of major depression in different groups of Latino older adults. The majority of the sample (56%) was Mexican American followed by Cuban Americans (13%), Puerto Ricans (8%), other (8%), or not specified (15%). They used the Composite International Diagnostic Interview—Short Form (CIDI-SF) to estimate the prevalence of major depression over the year preceding the interview (Steffick, 2000). The CIDI-SF major depression module is a structured lay interview keyed to criteria in the third edition of the Diagnostic and Statistical Manual (American Psychiatric Association, 1980). Yang et al. (2008) found that Puerto Ricans had the highest prevalence of major depression (19.3%), Mexican Americans had the lowest (8.2%), and Cuban Americans were in the middle (11.7%). It should be noted that the sample sizes were small (Mexican Americans = 427, Cuban Americans = 101, and Puerto Ricans = 56).

Recognizing that combining Latinos may be masking important intraethnic differences in major depression, González et al. (2010) compared the epidemiology of major depression within Latino subgroups across adulthood using the CPES data set. When looking at the older adult sample (aged older than 65 years), their results were similar to Yang et al. (2008). They found that Puerto Ricans had the highest 12-month and lifetime prevalence of major depression (10.2%, 18.4%), Mexican Americans had the lowest (4.6%, 9.0%), and Cuban Americans were in the middle (5.5%, 14.8%). González et al. (2010) did not solely focus on older adults, and when doing so, the sample sizes were small (Mexican Americans = 1,442, Cuban Americans = 577, and Puerto Ricans = 495). However, these results indicate that the homogeneity of the Latino population cannot be assumed, and when taken in conjunction with the HHANES and HRS results, they provide further evidence of differences in the expression and occurrence of depression among various Latino subgroups and provide support for presenting disaggregated data from Latino subgroups. Ignoring these important differences may yield erroneous and misleading findings (González et al., 2010). Table 1 summarizes findings from studies on depression and anxiety among older Latinos.

**Table 1.** Summary of the Prevalence of Depression and Anxiety Among Older Latinos

Authors	Sample	Age	Population	Key findings
Jimenez et al. (2010)	CPES	60+	Latinos (subgroup diverse)	Lifetime prevalence of any depressive disorder = 16.4%^a^
				Lifetime prevalence of any anxiety disorder = 15.3%^a^
				12-month prevalence of any depressive disorder = 8.0%^a^
				12-month prevalence of major depressive episodes = 7.3%^a^
Woodward et al. (2012)	CPES	55+	Latinos (subgroup diverse)	Lifetime prevalence of any depressive disorder = 13.9%^a^
				Lifetime prevalence of any anxiety disorder = 15.2%^a^
Mosciki et al. (1989)	HHANES	65–74	Mexican Americans	High depressive symptomatology = 13.2%^b^
Narrow et al. (1990)	HHANES	65–74	Cuban Americans	High depressive symptomatology = 11.3%^b^
Potter et al. (1995)	HHANES	65–74	Puerto Ricans	High depressive symptomatology = 27.9%^b^
Black et al. (1998)	H-EPESE	65+	Mexican Americans	High depressive symptomatology = 25.6%^b^
González et al. (2010)	SALSA	60–101	Predominately Mexican Americans	High depressive symptomatology = 25.4%^b^
Yang et al. (2008)	HRS	50+	Mexican Americans	Lifetime prevalence of major depression = 8.2%^c^
			Cuban Americans	Lifetime prevalence of major depression = 11.7%^c^
			Puerto Ricans	Lifetime prevalence of major depression = 19.3%^c^
González et al. (2010)	CPES	65+	Mexican Americans	Lifetime prevalence of major depression = 9.0%^a^
				12-month prevalence of major depression = 4.6%^a^
			Cuban Americans	Lifetime prevalence of major depression = 14.8%^a^
				12-month prevalence of major depression = 5.5%^a^
			Puerto Ricans	Lifetime prevalence of major depression = 18.4%^a^
				12-month prevalence of major depression = 10.2%^a^

*Note:* CPES = Collaborative Psychiatric Epidemiological Surveys; H-EPESE = Hispanic Established Populations for Epidemiologic Studies of the Elderly; HHANES = The Hispanic Health and Nutrition Examination Survey; HRS = Health and Retirement Study; SALSA = Sacramento Area Latino Study on Aging.

^a^As defined by the World Health Organization World Mental Health Composite International Diagnostic Interview (WMH-CIDI).

^b^As defined by a score of 16 or greater on the Center for Epidemiologic Studies Depression scale (CES-D).

^c^As defined by the Composite International Diagnostic Interview—Short Form (CIDI-SF).

## Immigration Histories and Sociopolitical Difficulties That Differentially Affect Mental Health

The U.S. Census Bureau (2011) defines Hispanic or Latino as an ethnicity that comprises individuals of Cuban, Dominican, Mexican, Puerto Rican, South or Central American, or other Spanish culture or origin regardless of race. Therefore, this is not a homogeneous, monolithic group, even though the U.S. Census definition classifies them into one uniform ethnicity. Although the majority of U.S. Latinos are of Mexican origin (33.7 million or 64% of the total U.S. Latino population in 2012), Latinos from Puerto Rico, Cuba, the Dominican Republic, and Central and South America account for a substantial proportion of Latinos living in the Eastern and Southern regions of the United States (Brown & Lopez, 2013). Despite this heterogeneity, national studies focusing on older Latino mental health often combine Latino subgroups into one, uniform group. Scientifically, this is done to have adequately powered studies. Culturally, there is some justification as there are many shared cultural characteristics and values such as language, familism, and religiosity (Alarcón, 2001; Gonzalez-Barrera & Lopez, 2013). However, despite sharing a common label, each Latino subgroup faces unique difficulties with regard to their immigration patterns and acculturation processes in the United States that differentially affect their mental health (Guarnaccia et al., 2005).

Mexican Americans are the youngest Latino subgroup: 35.9% are younger than the age 18, while 4.8% are 65 or older (U.S. Census Bureau, 2011). Approximately a third are foreign-born, and of these immigrants, it is estimated that 51% are undocumented (Gonzalez-Barrera & Lopez, 2013). Undocumented immigrants face numerous barriers such as lack of access to mental health care, insufficient financial resources, limited knowledge of the U.S. mental health care system, limited English proficiency, and increased stigmatization, all of which increase health risks (Derose et al., 2007). Also, the adverse circumstances under which many undocumented immigrants enter the United States, the substandard conditions in which many of them live and work, and the distress associated with living life without proper documentation are additional risk factors likely to diminish well-being among this immigrant subgroup (Cavazos-Rehg et al., 2007; Garcini et al., 2016). These immigrant-related factors and fear of deportation are unique to Mexican Americans compared to Puerto Ricans who are American citizens and Cuban Americans who receive political and legislative protection from the Cuban Readjustment Act (Gúzman & Carrasco, 2011).

Compared to all the Latino subgroups, Puerto Ricans are unique in that they are born U.S. citizens. This dual identity may contribute to a unique sense of dissonance and is an added stressor, which over time might explain the higher likelihood of psychological distress among Puerto Ricans (Alegria, Shrout, Woo, Guarnaccia, Sribney, Vila, Polo, Cao, Mulvaney-Day, Torres, & Canino, 2007; Guarnaccia et al., 2005). Puerto Ricans experience many social and economic inequalities that other Latinos do not experience. Despite being American citizens, Puerto Ricans experience the highest levels of everyday discrimination compared to Mexican and Cuban Americans (Molina et al., 2013). Against this backdrop, perhaps routine experiences of unfair treatment and inequality may heighten their attention to negative social information and experiences, which could potentially influence their mental health. The juxtaposition of Puerto Rican’s privileged status as American citizens while being economically and socially marginalized in U.S. society could potentially reverberate across generations (Guarnaccia et al., 2005).

Cuban Americans are the oldest of the Latino subgroups: 20.9% are younger than the age 18 while 16.1% are 65 or older (U.S. Census Bureau, 2011). The Cuban immigration experience is vastly different than that of any other Latino subgroup. Cuban immigrants were the only Latino immigrant group in the United States to receive political and legislative protection in the form of the Cuban Readjustment Act (Gúzman & Carrasco, 2011). This protection may have shielded them from discrimination and changed their experience of belonging and inclusion, at least among Cubans who immigrated closely following Castro’s assumption of power. Cubans report the lowest levels of everyday discrimination and experience distinct forms of privilege not afforded to other groups of Latinos, which may serve as buffers to the potential deleterious effects on mental health (Molina et al., 2013). Lower levels of discrimination are perhaps a function of the large numbers of Latinos living in Miami-Dade County which is 65% Latino, and Cubans comprise 54% of the Latino population (U.S. Census Bureau, 2012). Fewer opportunities for intergroup interaction in the community at large may reduce exposure to ethnicity-related discrimination (Molina et al., 2013). In addition, Cuban Americans tend to be the highest earners and have the greatest amount of economic resources than any other Latino subgroup (Alegria, Shrout, Woo, Guarnaccia, Sribney, Vila, Polo, Cao, Mulvaney-Day, Torres, & Canino, 2007; Suarez-Orozco & Paez, 2009). This increased socioeconomic status may in part be due to the Cuban Enclave Economy, which increases Cuban immigrant earnings and improves their chances of moving into self-employment (Portes, 1987; Waldinger, 1993). However, even this picture of Cuban Americans is complicated upon further inspection.

The 1980 Mariel event is seen as a line of demarcation in Cuban migration to the United States (Aguirre et al., 1997). In contrast to those who emigrated prior to 1980 who were largely viewed as righteous political dissidents, those who came after 1980 were depicted as the dregs of Cuban society by the Cuban government and the U.S. popular press (Hufker & Cavender, 1990; Portes & Stepick, 1993). U.S. migration policy toward Cuba was relatively unfavorable for the later arriving group, and they experienced increased discrimination and a greater likelihood of experiencing stressful life events in the United States, which are associated with declines in mental health (Ackerman et al., 2005; Cislo et al., 2010; González et al., 2005). Moreover, the conditions of migration between the two groups varied drastically. Earlier arrivals generally experienced relatively calm and safe passage (i.e., prearranged travel or through the Freedom Flights) while later arrivals endured more urgency in exiting and more hazardous passage (i.e., chartered boats of strangers or makeshift rafts; Campisi, 2005; Eckstein & Barberia, 2002; Portes & Stepick, 1993). Later arrivals became disconnected from family and friends, who were unable to exit or incapable of withstanding the harsh migration circumstances (Campisi, 2005). Thus, early and later arriving Cubans may have unequal levels of social support from family and friends, which can serve as coping resources to offset the deleterious effects of adversity and may translate into relatively poor health and adjustment outcomes for later arrivals (Cislo et al., 2010).

The healthy immigrant hypothesis, sometimes referred to as the immigrant paradox, posits that foreign nativity is protective against psychiatric disorders (Alegria, Shrout, Woo, Guarnaccia, Sribney, Vila, Polo, Cao, Mulvaney-Day, Torres, & Canino, 2007; Alegria et al., 2008). Epidemiologic studies have found that despite the stressful experiences and poverty often associated with immigration, Latino immigrants report lower rates of depressive, anxiety, and substance use disorders than U.S.-born Latinos and non-Latino whites (Alegria, Shrout, Woo, Guarnaccia, Sribney, Vila, Polo, Cao, Mulvaney-Day, Torres, & Canino, 2007; Alegria et al., 2008). However, when Latinos are disaggregated by age, subethnic group, and age of migration, a more complicated picture of Latino mental health emerges.

Findings from two studies using the CPES data set described above suggest that the healthy immigrant effects do not hold up in older age (González et al., 2010; Jimenez et al., 2010). Jimenez et al. (2010) found that older, immigrant Latinos (aged older than 60 years) had a higher lifetime prevalence of dysthymia and generalized anxiety disorder than U.S.-born Latinos. Similarly, results from González et al.’s study (2010) indicated that the prevalence of major depression was significantly higher for immigrant Latinos older than age 65 years compared to their U.S.-born counterparts. The results of these two studies suggest that immigrant health advantages earlier in life yield to the cumulative adversity of homeland estrangement, social isolation, and the overwhelming effects of socioeconomic disadvantages in later years when health needs and costs rise sharply (González et al., 2009, 2010). Moreover, older immigrant Latinos may lack the language and cultural fluency necessary to overcome social isolation and access barriers to quality mental health care that could relieve anxiety and depression (Alegria, Shrout, Woo, Guarnaccia, Sribney, Vila, Polo, Cao, Mulvaney-Day, Torres, & Canino, 2007). Age is not the only factor where there is inconsistency in the application of the healthy immigrant hypothesis. Further complicating the relationship between nativity and the risk for psychiatric illness is how the observed patterns may vary by age at arrival.

Latino immigrants who emigrate later in life (after age 35 years) experience high levels of psychosocial distress and are at high risk for developing a depressive disorder (Alegria, Shrout, Woo, Guarnaccia, Sribney, Vila, Polo, Cao, Mulvaney-Day, Torres, & Canino, 2007; Mills & Henretta, 2001). Late arrival may be protective in that it provides less opportunity for exposure to U.S. society, allowing for better retention of their native language and culture norms (Elder et al., 2005). However, these protective factors are overcome by the numerous difficulties associated with immigration late in life. Migration negatively affects traditional Latino cultural values (e.g., *familism, respeto*) that might otherwise serve to buffer stressful life circumstances linked to an increased risk of psychiatric disorders (Almeida et al., 2009; Campos et al., 2008; Jimenez, Bartels, Cardenas, Dhaliwal, & Alegria, 2012). For later arrival immigrants, the disruption of these cultural values might result in isolation and decreased family and social support, which are exacerbated by low English proficiency. Older Latino immigrants who have not yet mastered the English language come to rely on their children who have gained greater English language proficiency through schooling and interactions with the mainstream culture. This may lead to role reversals, where their children take a more authoritative role, which may lead to regret and disappointment in later arrival immigrants and erode the affiliative obedience and respect for older adults (Elder et al., 2005).

Additional difficulties such as educational or professional qualifications not translating to the host society and being a minority in the United States can help to explain further the increased risk of depressive disorders in later arrival immigrants. They might not benefit from the same level of achievement as their younger counterparts nor reap the same rewards of their hard work. Also, later arrival immigrants may become increasingly aware of ethnic and racial categories used by the U.S. society which seem unfair and pejorative, particularly when they have an already ingrained identity coming from the majority culture. This may increase the risk of psychopathology, as they may experience many years of living in the United States while not fully integrated into U.S. society (Alegria, Shrout, Woo, Guarnaccia, Sribney, Vila, Polo, Cao, Mulvaney-Day, Torres, & Canino, 2007).

## Risk and Protective Factors

Older Latinos, in general, have identified a social support network as an essential component to mental health (Jimenez, Bartels, Cardenas, Dhaliwal, & Alegria, 2012). The social support of family and close friends is considered to exert a positive impact on physical and mental health and these healthful effects of social connection are primarily experienced through individual-level support (e.g., family and friends; Alegria, Sribney, & Mulvaney-Day, 2007; Campos et al., 2008). However, once the social support network is disrupted by immigration to the United States, these benefits may be quickly lost. The effect of the scattering of family members and close friends on family structure and relationships is believed to be traumatic and can lead to poor mental health (Jimenez, Bartels, Cardenas, Dhaliwal, & Alegria, 2012). This tearing of the social fabric brings and can lead to a detrimental loss of belonging, social isolation, and increased intergenerational conflict (Almeida et al., 2009; Campos et al., 2008; Elder et al., 2005).

Discrimination is often seen as a pathogen that generates depression among people of color (Fernando, 1984). Specifically, it has been found that the relation between discrimination and health among Latinos is mediated by psychological distress, and that this effect is stronger for Latinos than for other racial/ethnic groups (Brondolo et al., 2011). Finch et al. (2000) found that in a sample of Mexican Americans, perceived discrimination was associated with increased levels of psychological distress. Furthermore, the closer the perceived discrimination is to one’s self, the psychological distress increases (Jackson et al., 2006). Jointly, this suggests that the effect of discrimination on psychological distress may be augmented for Puerto Ricans, who by account of their multiple stigmatized social identities—American citizens who are economically and socially marginalized in U.S. society—evidence higher levels of unfair treatment and other social stressors compared to their Latino counterparts (Molina et al., 2013).

## Meeting the Mental Health Needs of Older Latinos

In order to address the mental health needs of older Latinos, it is important to keep in mind the considerable variability between Latino subgroups, which highlights the need for cultural case conceptualizations of individual patients and to tailor interventions to the unique circumstances and backgrounds of the patient. For example, in the original cognitive behavioral therapy (CBT) model, patients were taught techniques to “control” their depression by changing behaviors and thoughts (Lewinsohn et al., 1978). Muñoz (1996) realized that this technique had limited contextual relevance for the predominately Mexican patients he was treating in San Francisco because there are particular realities that low-income, uninsured Mexican individuals do not have control over (e.g., the type of neighborhood they live in, and experiences of discrimination, racism, and poverty). This led him to adapt the CBT approach to focus on the healthy management of reality (Muñoz, 1996). At the same time, it is necessary to consider the extent to which common elements and interventions can generalize across Latino cultures. We have found that certain cultural values and experiences (e.g., importance of family, language, shared ancestry, and religion) play a powerful role across multiple Latino subgroups. The realities of clinical practice or clinical research in a public sector setting often mandate the inclusion of members from varied cultural backgrounds within a single intervention group. The awareness and explicit acknowledgment of cultural similarities and differences are very important in such a context.

Many evidence-based treatments have been adapted for Latino communities to address the cultural and contextual realities of the Latino community, which may influence service utilization and intervention outcomes (Comas-Díaz, 2006; Whaley & Davis, 2007). Further tailoring is done in order to ensure the treatment is compatible with the individual’s background, age, migration history, and cultural meanings and values (Bernal et al., 2009). One of the most studied evidence-based treatments that has been adapted for older Latinos is CBT. Although no empirical evidence suggests that Latinos respond more favorably to CBT than other forms of psychotherapy, certain dimensions of CBT are consistent with the traditional cultural characteristics and social experiences of older Latinos (Organista, 2006). First, CBT’s emphasis on education frames life as an instructive experience whereby one learns and teaches existential lessons (Muñoz & Mendelson, 2005). Cultural attitudes about mental illness and therapy confer blame and stigma within Latino communities (McCabe et al., 2005). An educational and didactic approach along with the use of patient manuals and in-home assignments helps older Latinos view therapy as more of a classroom experience, thus decreasing the stigma so often attached to therapy (McCabe et al., 2005; Organista, 2006). Second, CBT is present focused and emphasizes problem solving, thus providing concrete solutions and techniques used to solve such problems. For example, the CBT technique of challenging and changing negative thoughts is potentially congruent with Latino cultural resilience (Comas-Díaz, 2006).

Despite the cultural adaptations and the effectiveness of CBT and other evidence-based treatments, older Latinos, as a whole, have limited access to these treatments and, for reasons of stigma, are often reluctant to engage in help seeking (Jimenez, Bartels, Cardenas, & Alegria, 2012; Jimenez et al., 2013). Furthermore, available mental health treatments may not match the preferences and beliefs of older Latinos, which can lead to the decision to not access mental health treatment (Jimenez, Bartels, Cardenas, Dhaliwal, & Alegria, 2012). Therefore, mental health treatments do not sufficiently reduce the prevalence of depression or anxiety in older Latinos, thus underscoring the importance of prevention and addressing an urgent and rapidly growing medical and public health issue.

Over the last 25 years, the number of randomized controlled trials testing preventive interventions has greatly increased (Muñoz, 2019). A meta-analysis of 32 studies in 2014 showed that in all sorts of groups that are at risk—from expectant and new mothers to individuals who had experienced a stroke—such preventive interventions reduce the onset of major depressive episodes by 21%, on average (van Zoonen et al., 2014). In the same year, Muñoz et al. (2014) found that 15 of 42 randomized trials reported reductions of 50% or more in the incidence of depression. Moreover, depression prevention interventions consistently report effect sizes similar to those for the use of statins to prevent acute myocardial infarction during a 5-year period (van Zoonen et al., 2014). In short, the data suggest that if we implement effective interventions early on, then we could reduce new cases of depression in half (Muñoz, 2019). While these meta-analyses show robust evidence of the effectiveness of prevention interventions as a whole, they do not tell us the effect of these interventions on Latinos, specifically older Latinos.

One of the earliest prevention interventions to target depression prevention in the Latino community was the Depression Prevention Course (DPC) developed by Muñoz (1984). Social learning theory (SLT; Bandura, 1977) was the basis for the DPC because the theory embodies principles of behavior change that are universally relevant and potentially applicable across cultures (Muñoz & Mendelsson, 2005). The DPC was designed to teach mood management skills to primary care patients in order to prevent major depressive episodes and was evaluated in a randomized controlled trial at San Francisco General Hospital with 150 primary care patients. Twenty-four percent of the sample consisted of Latinos, predominately of Mexican descent. The DPC was translated into Spanish and underwent a lengthy cultural adaptation process in order to account for the particular realities that low-income, ethnic minority, uninsured individuals do not have control over (e.g., the type of neighborhood they live in, and experiences of discrimination, racism, and poverty). In addition, the tone of the intervention was consciously skills building and learning oriented. The DPC proved to be effective in significantly reducing depressive symptoms and the number of new episodes of major depression was lower in the experimental condition at 1-year follow-up compared to the control condition (Muñoz & Ying, 1993; Muñoz et al., 1995).

Although the DPC did not specifically target Latinos, the overall positive impact in reducing depression in this population has encouraged researchers to replicate and evaluate the intervention in various ways. It has been adapted for pregnant Latinas at high risk for postpartum depression in San Francisco (predominately Mexican descent) and Washington, DC (predominately Central American descent), adolescent depression in Puerto Rico, and low-income Latinas (subgroup diverse; Le et al., 2004, 2011; Miranda et al., 2003; Rosselló et al., 2008). Despite the increased focus on prevention and the success of the DPC in the Latino community, few prevention interventions have focused on older Latinos. Given the prevalence and morbidity of depression and anxiety in later life, the inadequacies of current treatment approaches for averting years living with a disability, the disparities in access to the mental health care delivery system, and the workforce shortages to meet the mental health needs of older Latinos, development and testing of innovative strategies are needed to stop depression and anxiety from taking hold in the first place.

In an attempt to address the dearth of prevention research in older Latinos, the Happy Older Latinos are Active (HOLA; Jimenez, Reynolds et al., 2015) intervention was created. HOLA is a community health worker (CHW)-led, multicomponent, health promotion intervention consisting of (a) two manualized social and physical activation sessions; (b) a moderately intense group walk led by a CHW for 45 min, 3×/week for 16 weeks; (c) pleasant events scheduling; and (d) a maintenance phase consisting of one “booster” walking session twice a month for 6 months postintervention, then once a month for 18 months after that in order to reinforce the behavior change and maintain the effects of the intervention. Similar to the DPC, HOLA is based on SLT, but diverges from the DPC in that it incorporates the principles of Behavioral Activation to engage older Latinos in a physical activity routine as well as pleasant events scheduling to reduce the risk for major depression and generalized anxiety disorder, prevent incidence and recurrence of these disorders, and improve health-related outcomes. HOLA was pilot tested in a group of diverse older Latinos. The intervention was culturally tailored based on participant feedback that directly contributed to the features, components, and delivery of the intervention. Therefore, it is believed that it will appeal to all Latino subgroups.

Preliminary results from the pilot study indicate that HOLA is feasible and acceptable and highlights the significant impact the intervention can have in lowering depression and anxiety vulnerability when it is relevant, respectful, and specific to the needs of the older Latino population (Jimenez et al., 2018). A randomized prevention trial to examine the effectiveness of HOLA in reducing the risk for and incidence/recurrence of major depression and generalized anxiety disorder in a diverse sample of older Latinos is currently under way (NCT03870360). If effective, HOLA can be explicitly linked to preventing depression and anxiety in older Latinos and rapidly disseminated as a prevention intervention throughout the country at a low cost. In the context of reductions in funding available for preventive health services, particularly within mental health, this study may exemplify the use of health promotion with minimal use of resources.

## Future Directions for Mental Health Research Among Older Latinos

Consideration of the heterogeneity of the older Latino population is critical for the advancement of mental health research in this segment of the population. While the present review has focused on differences among Latinos subgroups based on country of origin/descent and age, other factors such as sex/gender, acculturation/language use, and geographical region of residence in the United States should also be considered in understanding mental health determinants among older Latinos, and in the development and implementation of mental health prevention and intervention approaches for this diverse group. In addition, there are likely other sociocultural determinants important for the mental health of older Latinos that are more difficult to quantify, such as the immigration experience and early childhood environment, among others. All these factors are thought to be important for understanding mental health among older Latinos. However, we do not have a full understanding of which are the most important ones; how they interact to affect mental health among older Latinos; and how we can accurately assess them. Notably, mental health research among Latinos cannot be detached from the sociocultural zeitgeist and historical events. It is essential that older Latinos continue to be part of the scientific dialogue related to mental health care needs and best practices. This is especially true given recent immigration and health policies that have negatively affected the lives of many Latinos in the United States, with some subgroups of this population being more affected than others (Hatzenbuehler et al., 2017). Furthermore, the current COVID-19 pandemic is disproportionally affecting Latinos and other under-represented groups in the United States (Centers for Disease Control and Prevention [CDC], 2020a, 2020b). The impact that these factors have on the mental health of the older Latino population in the United States is yet to be elucidated. Longitudinal studies of mental health among diverse Latinos subgroups will be better equipped to understand mental health and its determinants among older Latinos.

## Conclusions

The aggregation of Latino subgroups in national studies creates an overly simplistic narrative that Latinos are at lower risk of mental illness and that foreign nativity seems protective against mental illness (i.e., immigrant paradox). This broad generalization masks key differences in the prevalence of depression and anxiety, immigration histories, and sociopolitical difficulties that differentially affect their mental health. As the older Latino population continues to grow, it will be important to understand how these determinants differentially affect not only the three Latino subgroups with the longest residential history in the United States (Mexicans, Puerto Ricans, and Cubans) but other subgroups whose populations are expected to rise as well (Dominicans, Colombians, Venezuelans, etc.). Other factors such as sex/gender, acculturation/language use, and geographical region of residence in the United States should also be considered in understanding mental health determinants among older Latinos and in the development and implementation of intervention approaches.

Nativity alone does not protect from psychiatric illness once immigrants arrive as originally thought. Rather, it is a complex combination of family, contextual, and social status factors associated with nativity and age of arrival in the United States that has the protective effect (Alegria, Shrout, Woo, Guarnaccia, Sribney, Vila, Polo, Cao, Mulvaney-Day, Torres, & Canino, 2007). These factors signal critical windows of opportunity for addressing differential risks for older Latinos and pathways to intervene in order to prevent negative outcomes.

Although numerous treatments for depression and anxiety are effective and available, many older Latinos are not accessing these services due to structural (e.g., language, income, insurance, and provider shortage) and psychological (e.g., stigma) barriers. These challenges combined with the scale of the problem require strategies to stop depression and anxiety from taking hold in the first place. The development of depression and anxiety prevention strategies would be a means of addressing multiple inequalities in older Latino mental health. Culturally tailored depression prevention tools have been developed and implemented successfully for younger Latinos across the United States. However, work remains to be done to demonstrate the longer-term sustainability of such prevention strategies in older Latinos. Success in this endeavor would yield a substantial reduction in the burden of late-life depression and anxiety and has the potential to change practice.
